# Supplementary material for: A Comprehensive Assessment of the Genetic Determinants in Salmonella Typhimurium for Resistance to Hydrogen Peroxide Using Proteogenomics
Source: Sci Rep. 2017 Dec 6;7:17073. doi: 10.1038/s41598-017-17149-9 (PMC5719062; doi:10.1038/s41598-017-17149-9)
Supplement: Supplementary file 1 — Supplementary Information [file 41598_2017_17149_MOESM1_ESM.pdf]

Supplementary information

A Comprehensive Assessment of the Genetic Determinants in *Salmonella* Typhimurium for Resistance to Hydrogen Peroxide Using Proteogenomics

Sardar Karash[1], Rohana Liyanage[2,3], Abdullah Qassab[1], Jackson O. Lay, Jr.[2,3], and Young Min Kwon[1,4*]

[1]Cell and Molecular Biology Program, University of Arkansas, Fayetteville, AR 72701,

[2]Department of Chemistry, University of Arkansas, Fayetteville, AR 72701,

[3]Statewide Mass Spectrometry Facility, Fayetteville, AR 72701, and

[4]Department of Poultry Science, University of Arkansas, Fayetteville, AR 72701

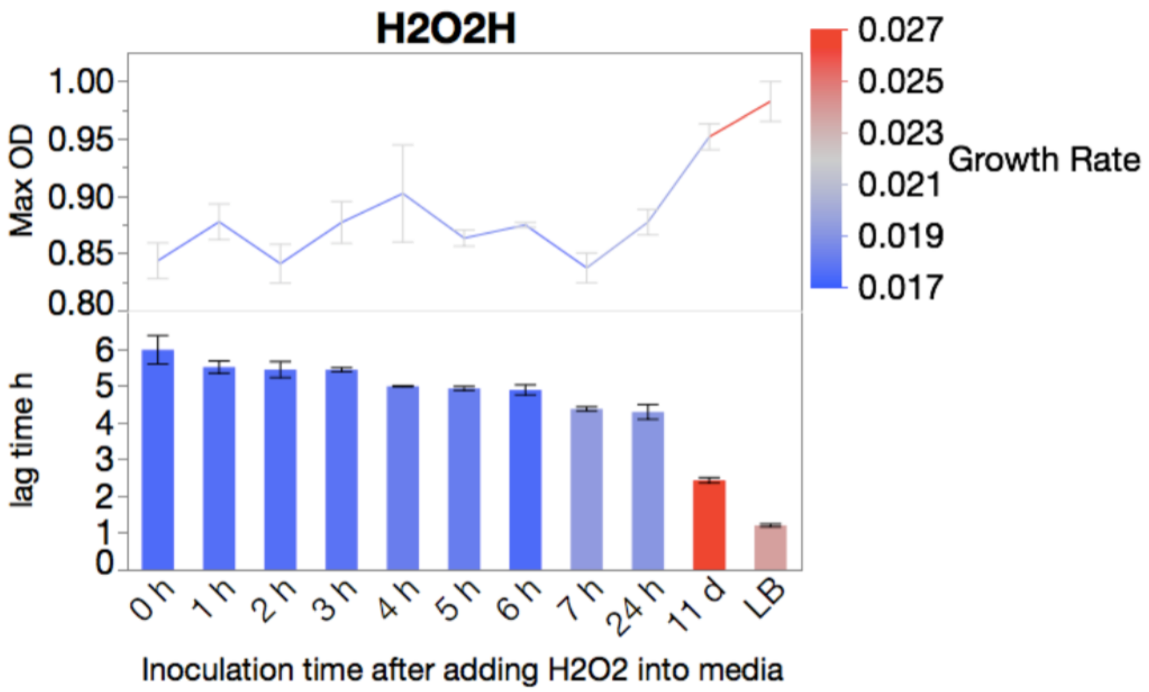

Figure S1. Stability of H_2O_2 in LB medium during the experiments. LB broth media supplemented with freshly diluted 3.5 mM H_2O_2 were left at room temperature for the indicated lengths of time before inoculation of the media with *S.* Typhimurium. The inoculated cultures were incubated at 37°C for 24 h in a 96-well plate with OD_{600} reading every 10 minutes, and lag time and Max OD were determined. LB media free of H_2O_2 was used as a control.

	Number of extracted GACAG_reads >20bp	Number of Mapped Reads	Number of Unique Insertions	Mean genomic length bp	Mean reads per insertion sites	Mean distance between two adjacent insertions	Number of reads mapped to pBAM-1	% of pBAM1
**LB**	31728005	25223444	125449	92.9	201.0653254	38.822661	181359	0.571605432
**H2O2L**	17986240	14855156	118169	93.3	125.7111087	41.21439633	109506	0.608832085
**H2O2H**	22665911	18622648	119801	94.6	155.4465155	40.65294947	80421	0.354810358
**Total**	72380156	58701248	363419				371286	1.535247875
**Average**				93.6	160.7409832	40.23000227	123762	0.511749292
**STD**				0.888819442	37.95507916	1.250704891	51957.14689	0.137181732

Table S1. Summary of the Tn-seq analysis in numbers. The numbers of extracted reads, mapped reads, and unique insertions are presented for LB (H_2O_2 free), H_2O_2L (2.5 mM), and H_2O_2H (3.5 mM). Mean reads per insertion sites were calculated by dividing number of mapped reads by number of unique insertions, and mean distance between two adjacent insertions was calculated by dividing the length of whole genome by number of unique insertions. Reads mapped to pBAM1 represent the mutants generated by cointegration of pBAM1.

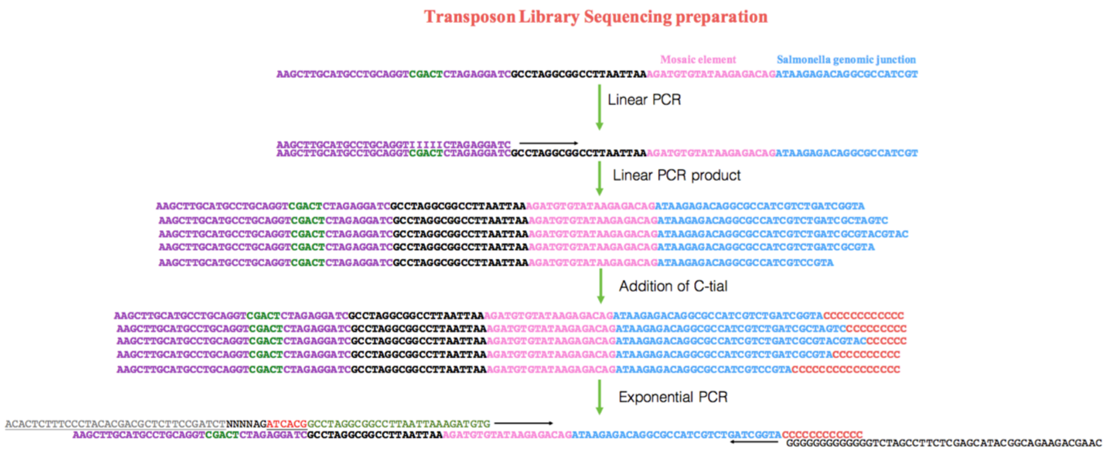

Figure S2. Tn-seq library preparation for Illumina sequencing. The genomic DNA was extracted from each mutant pool, and then subjected to the protocol as illustrated here. First step was linear extension using a single primer specific to one end of Tn5 to capture Tn5 junctions. The second step was to add C-tail at 3’ end of the captured Tn5 junction fragments.

Lastly, the C-tailed Tn5 junction fragments were used as a template for exponential PCR using nested Tn5-specific primer and polyG primer with Illumina adaptor and a sample barcode attached. The PCR product was gel-purified and sequenced on an Illumina platform.

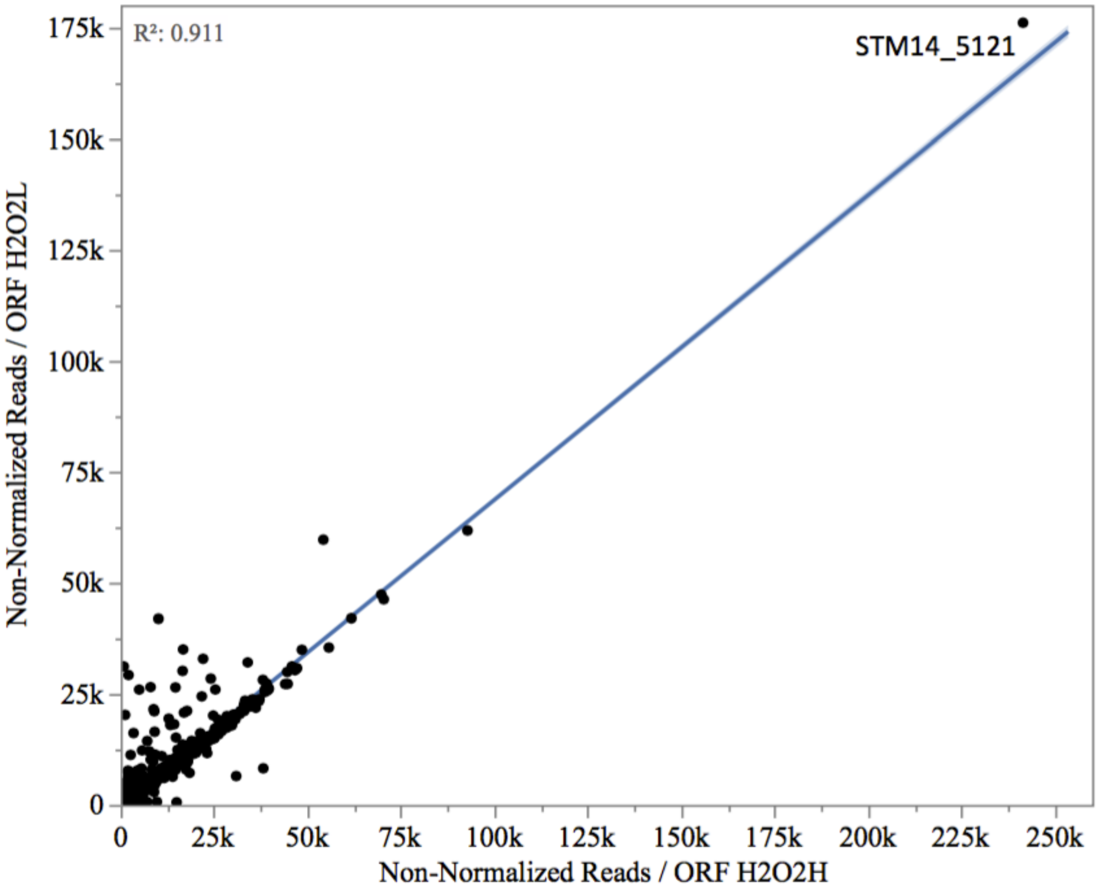

Figure S3. The reproducibility of Tn-seq. Correlation of the number of reads for each ORFs in *S.* Typhimurium between H_2O_2L (2.5 mM) and H_2O_2H (3.5 mM). Two ORFs (STM14_2422 and STM14_2428) were excluded in this analysis.

The enriched pathways for resistance to H_2O_2

In order to categorize the identified genes that are required for *Salmonella* resistance to H_2O_2, the 137 genes were subjected to pathway enrichment analysis using DAVID Bioinformatics Resources 6.7, NIAID/NIH[31]. A total of 15 KEGG pathways[32] were recognized for 69 genes on the list. The enriched pathways include homologous recombination (*ruvC*, *polA*, *ruvA*, *ruvB*, *priB*, *recA*, *recR*, *holC*, *holD*, *recC*, *recG*), nucleotide excision repair (*uvrD*, *polA*, *uvrA*, *uvrC*), mismatch repair (*dam*, *uvrD*, *holC*, *holD*), RNA degradation (*pnp*, *hfq*, *ygdP*), purine and pyrimidine metabolism (*apaH*, *polA*, *pnp*, *arcC*, *spoT*, *holC*, *holD*, *cmk*, *dcd*, *pnp*), phenylalanine, tyrosine and tryptophan biosynthesis (*aroD*, *aroB*, aroA, *aroK*, *aroE_2*), arginine and proline metabolism (*proC*, *arcC*), glycolysis and gluconeogenesis (*crr*, *pgm*, *tpiA*), oxidative phosphorylation (*atpG*, *atpA*, *cydA*), DNA replication (*polA*, *holC*, *holD*), and flagellar assembly (*fliJ*, *fliD*, *flhD*, *fliC*). Since KEGG was not able to recognize many genes on the list, we used SP_PIR_Keywords of functional categories, which recognized majority of the genes and categorized them into 55 functional categories (Table S3), excluding 15 uncharacterized genes (ORFs). Among these categories were stress response (*rpoE*, *lon*, *dnaJ*, *hfq*, *yaiB*), iron (*dps*, *entD*, *iscA*, *yjeB*, *yhgI*), and transcription regulation (*rcsA*, *oxyR*, *rpoE*, *yjeB*, *arcA*, *argR*, *rbsR*, *rpoS*, *fadR*, *rcsB*, *furR*, *flhD*).

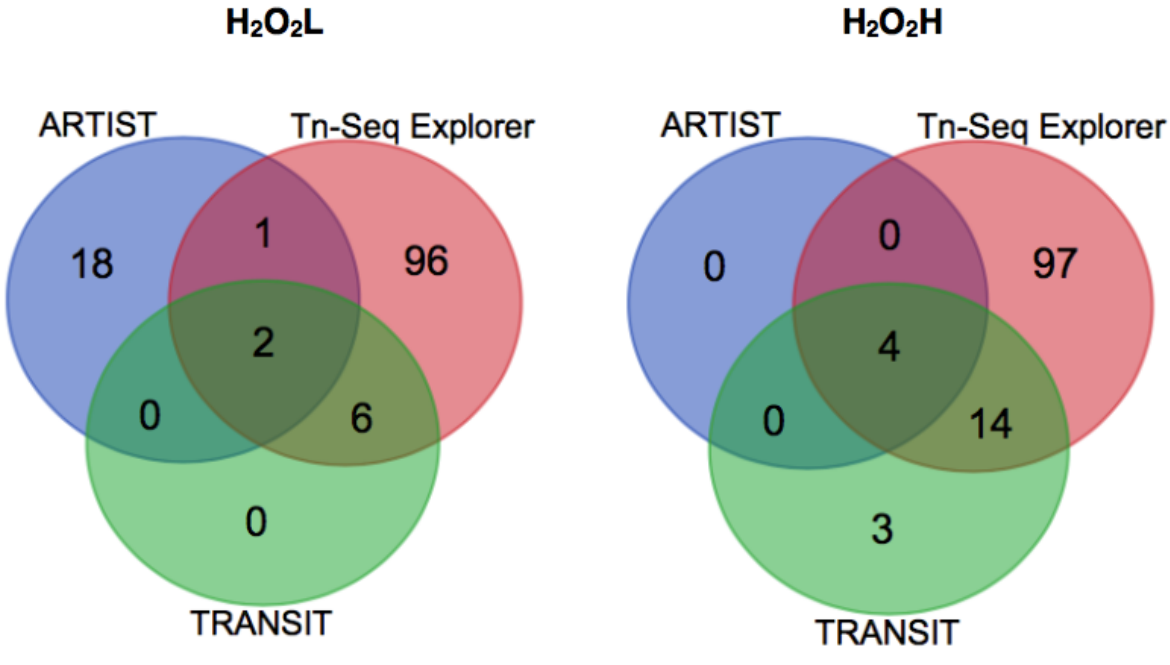

Figure S4. Comparison of various bioinformatics pipelines for Tn-seq data analysis. The Tn5 mutant library was grown till mid-log phase *in vitro* in LB media containing either 2.5 mM H_2O_2 (H_2O_2L) or 3.5 mM H_2O_2 (H_2O_2H). Three different tools were used for analysis of resulting Tn-seq data: ARTIST, Tn-seq Explorer, and TRANSIT.

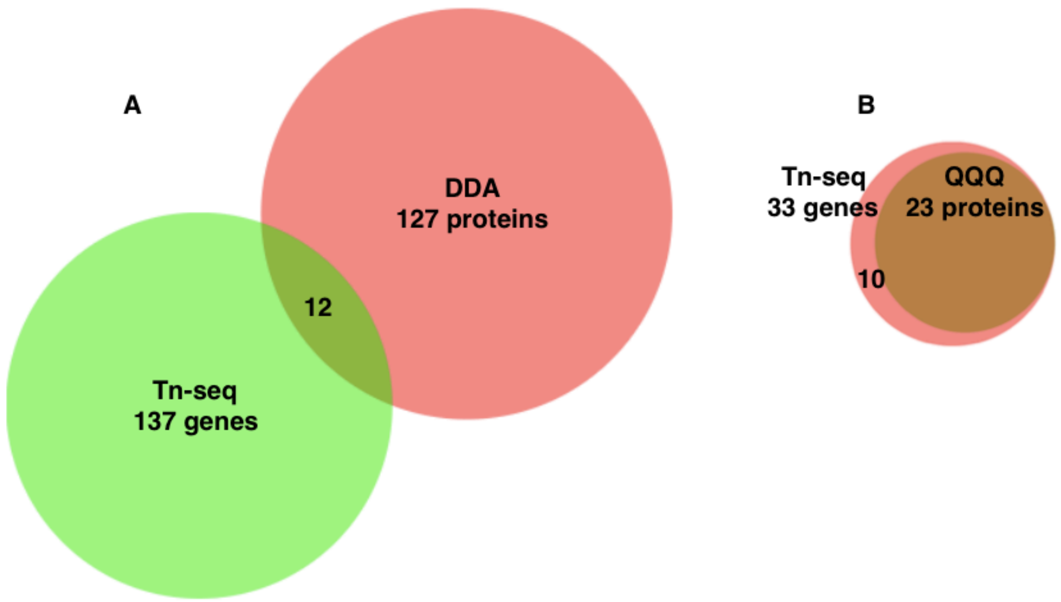

Figure S5. Overlapping genes/proteins identified by Tn-seq and proteomics approaches. The Tn5 mutant library was grown till mid-log phase in the presence of two different H_2O_2 for Tn-seq. The wild type strain was grown till mid-log phase for proteomics. (A) DDA (data dependent analysis) was used for proteomic analysis. The number of upregulated proteins in response to H_2O_2 was 127, $p < 0.05$. The number of conditional essential genes for H_2O_2 resistance identified by Tn-seq was 137. Overlapped genes/proteins by both methods were 12. (B) A total of 33 conditionally essential genes identified by Tn-seq were targeted in the proteomic analysis using QQQ (quadrupole mass spectrometry). The number of targeted proteins upregulated in response to H_2O_2 was 23, $p < 0.05$.

Functional Categories	Count	%	Genes
dna repair	10	7.8125	*ruvB, uvrC, ruvA, uvrD, uvrA, xthA, recR, ruvC, recA, polA*
DNA damage	10	7.8125	*ruvB, uvrC, ruvA, uvrD, uvrA, xthA, recR, ruvC, recA, polA*
cytoplasm	22	17.1875	*gidA, tpiA, uvrC, crr, uvrA, xerC, xerD, cmk, recA, gmhA, pnp, dps, argR, sufS, aroK, efP, fadR, aroB, dnaJ, aroA, yaiB, flhD*
dna recombination	7	5.46875	*ruvB, ruvA, xerC, xerD, recR, ruvC, recA*
sos response	6	4.6875	*ruvB, uvrC, ruvA, uvrD, uvrA, recA*
atp-binding	20	15.625	*atpA, sufC, ruvB, lon, uvrD, ruvA, uvrA, cmk, yjeA, recA, barA, fepC, recG, hscA, ybbL, aroK, arcB, phoL, phoR*
hydrolase	21	16.40625	*atpA, dcd, lon, ruvB, uvrD, ruvA, recC, hutG, rnt, xthA, polA, arcA, recG, apaH, yejM, endA, lepB, degS, ruvC, ygdP, spoT*
nucleotide-binding	19	14.84375	*atpA, sufC, lon, ruvB, uvrD, ruvA, uvrA, cmk, yjeA, recA, barA, fepC, recG, hscA, ybbL, aroK, arcB, phoR*
dna-binding	23	17.96875	*rcsA, rpoE, oxyR, lon, uvrD, ruvA, uvrA, xerC, xerD, yjeB, recA, acrR, arcA, polA, dps, argR, rbsR, rpoS, fadR, rcsB, fruR, priB, flhD*
metal-binding	16	12.5	*entD, iscA, uvrA, icdA, hutG, xthA, yjeB, dps, aroK, dksA, ruvC, recR, dnaJ, yhgI, pgm*
aromatic amino acid biosynthesis	5	3.90625	*aroK, aroE, aroD, aroB, aroA*
stress response	6	4.6875	*rpoE, lon, dnaJ, hfq, yaiB*
signal	14	10.9375	*yejE, fepD, barA, yejM, endA, mrdA, arcB, ybbM, degS, phoR, sthB, cysP, ompS, cbiM*
amino-acid biosynthesis	7	5.46875	*argR, aroK, aroA, aroD, aroB, aroA, proC*
DNA binding	4	3.125	*rcsA, argR, rpoS, fruR*
zinc-finger	4	3.125	*uvrA, dksA, recR, dnaJ*
transferase	20	15.625	*ybaZ, fliB, rfaF, entD, crr, slrB, cmk, barA, polA, pnp, otsA, sufS, aroK, arcC, holD, holC, arcB, dam, phoR, aroA*
dna replication	5	3.90625	*uvrD, dam, pola, dnaj, prib*
activator	5	3.90625	*rcsA, fadR, rcsB, fruR, flhD*
exonuclease	4	3.125	*rnt, recC, xthA, polA*
transport	14	10.9375	*atpA, entD, crr, yejE, fepD, corA, tonB, atpG, sapC, fliJ, exbD, sthB, cysP, ompS*
protein transport	4	3.125	*sapC, fliJ, exbD, tonB*
nuclease	4	3.125	*rnt, xthA, ruvC, polA*
magnesium	6	4.6875	*aroK, entD, xthA, corA, ruvC, pgm*
flagellum	4	3.125	*fliJ, fliD, fliC, flhD*
repressor	4	3.125	*argR, fadR, yjeB, fruR*
phosphoprotein	5	3.90625	*arcB, crr, rcsB, phoA, barA*
helicase	4	3.125	*ruvB, ruvA, uvrD, recG*
nucleotidyltransferase	4	3.125	*holD, holC, polA, pnp*
ion transport	5	3.90625	*atpaA, entD, corA, ompS, atpG*
iron	6	4.6875	*dps, entD, iscA, yjeB, yhgI*
Isomerase	6	4.6875	*slpA, rpe, tpiA, gmhA, pgm*
excision nuclease	2	1.5625	*uvrC, uvrA*
capsule biogenesis/ degradation	2	1.5625	*rcsA, rcsB*
transcription regulation	13	10.15625	*rcsA, oxyR, rpoE, yjeB, acrR, arcA, argR, rbsR, rpoS, fadR, rcsB, fruR, flhD*
Transcription	13	10.15625	*rcsA, oxyR, rpoE, yjeB, acrR, arcA, argR, rbsR, rpoS, fadR, rcsB, fruR, flhD*
dna integration	2	1.5625	*xerC, xerD*
Chromosome partition	2	1.5625	*xerC, xerD*
Chaperone	4	3.125	*sthB, dnaJ, hscB, hscA*
kinase	7	5.46875	*aroK, arcC, arcB, crr, cmk, phoR, barA*
trna processing	3	2.34375	*gidA, rnt, yhdG*
cell membrane	12	9.375	*atpA, rpe, yejE, lepB, sapC, fliJ, fepD, corA, tonB, ompS, yejM, atpG*
membrane	13	10.15625	*atpA, rpe, yejE, lepB, sapC, fliJ, fepD, corA, sthB, tonB, ompS, yejM, atpG*
dna excision	2	1.5625	*uvrC, uvrA*
nadp	3	2.34375	*icdA, aroA, proC*
cf(1)	2	1.5625	*atpA, atpG*
Protease	4	3.125	*lon, degS, lepB, araH*
cell inner membrane	7	5.46875	*atpA, lepA, sapC, corA, tonB, yejM, atpG*
two-component regulatory system	4	3.125	*arcB, rcsB, phoA, barA*
Sigma factor	2	1.5625	*rpoE, rpoS*
rna-binding	3	2.34375	*hfq, pnp*
bacterial flagellum biogenesis	2	1.5625	*fliJ, flhD*
bacterial flagellum	2	1.5625	*fliD, fliC*
atp synthesis	2	1.5625	*atpA, atpG*
Hydrogen ion transport	2	1.5625	*atpA, atpG*
Unknown ORF	15		*STM14_0196, STM14_1174, STM14_1758, STM14_1944, STM14_2358, STM14_2430, STM14_3007, STM14_3217, STM14_3219, STM14_3285, STM14_5452, STM14_5491, STM14_5494, STM14_5495, STM14_5517*

Table S3. Functional categories for the 137 genes conditionally essential for *S.* Typhimurium resistance to H_2O_2. SP_PIR_Keywords were used with default options for functional categories analysis for all 137 conditionally essential genes identified in this study. The gene recognition by the analysis tool was based on official gene symbols.

Pathways	Proteins
**Ribosome**	**RplB**, **RplC**, **RplD**, **RplE**, **RplI**, **RplJ**, **RplK**, **RplL**, **RplM**, **RplN**, **RplO**, **RplP**, **RplQ**, **RplR**, **RplT**, **RplU**, **RplV**, **RplW**, **RplX**, **RpmA**, **RpmD**, **RpmI**, **RpoA**, **RpoC**, **RpsA**, **RpsB**, **RpsC**, **RpsD**, **RpsE**, **RpsG**, **RpsH**, **RpsI**, **RpsJ**, **RpsK**, **RpsL**, **RpsM**, **RpsO**, **RpsS**, **RpsU**
**Glycolysis / Gluconeogenesis**	AdhP, Acs, **AceF**, **Eno**, AceE, PykF, Crr, **GpmA**, FbaB, **LpdA**, GlpX, Pgm, PfkB, **Pgk**
**Pyruvate metabolism**	Acs, **AceF**, AceE, PykF, **AccB**, Mdh, Pta, Ppc, LldD, **LpdA**, GloA, **PflB**
**Pentose phosphate pathway**	**PrsA**, TalB, FbaB, DeoB, RpiA, Eda, GlpX, Pgm, PfkB
**Citrate cycle (TCA cycle)**	SucB, **AceF**, AceE, AcnB, IcdA, Mdh, **LpdA**, GltA
**Amino sugar and nucleotide sugar metabolism**	GalK, ManA, Crr, GalT, GalE, YfbG, ManX, Pgm, NagZ
**Propanoate metabolism**	Acs, **AccB**, Pta, PrpE, PflB, PrpC
**Fatty acid biosynthesis**	**FabI**, **FabG**, **AccB**, FabF, **FabB**
**Purine metabolism**	**PrsA**, GuaB, PykF, **RpoC**, **Adk**, **RpoA**, Hpt, Ndk, DeoB
**Galactose metabolism**	GalK, GalT, GalE, Pgm, PfkB
**Streptomycin biosynthesis**	RfbA, Pgm, RfbD
**Riboflavin metabolism**	**RibH**, PhoN
**Fructose and mannose metabolism**	ManA, FbaB, GlpX, ManX, PfkB
**Glyoxylate and dicarboxylate metabolism**	AcnB, Mdh, Eda, GltA
**Pyrimidine metabolism**	Upp, **RpoC**, PyrC, **RpoA**, Ndk
**Glycine, serine and threonine metabolism**	**Asd**, GcvP, **LpdA**, GlyA
**RNA degradation**	GroEL, **Eno**, **Rho**
**Two-component system**	PhoN, ArcB, GlnB, CpxR, PhoP, ArcA, FliC
**gamma-Hexachlorocyclohexane degradation**	PhoN, DlhH
**Fructose and mannose metabolism**	ManA, GlpX, ManX, PfkB
**RNA polymerase**	**RpoC**, **RpoA**

Red are upregulated proteins, blue are downregulated, and **bold** are essential proteins/genes (based on the Tn-seq).

Table S6. Deferentially expressed proteins of *S.* Typhimurium in response to H_2O_2 and their pathways. *S*. Typhimurium wild type strain was grown in LB (H_2O_2 free), H_2O_2L (2.5 mM), or H_2O_2H (3.5 mM) till mid-log phase. KEGG pathway analysis was used to categorize deferentially expressed proteins ($p < 0.05$). Blue for downregulated proteins, red for upregulated proteins and bold represents essential proteins.
